# Important considerations regarding the widespread use of doxycycline chemoprophylaxis against sexually transmitted infections

**DOI:** 10.1093/jac/dkad129

**Published:** 2023-05-02

**Authors:** Fabian Yuh Shiong Kong, Chris Kenyon, Magnus Unemo

**Affiliations:** Centre for Epidemiology and Biostatistics, Melbourne School of Population and Global Health, University of Melbourne, Melbourne, Australia; HIV/STI Unit, Institute of Tropical Medicine, Antwerp, Belgium; Division of Infectious Diseases and HIV Medicine, University of Cape Town, Cape Town, South Africa; WHO Collaborating Centre for Gonorrhoea and Other STIs, National Reference Laboratory for STIs, Department of Laboratory Medicine, Örebro University, Örebro, Sweden; Faculty of Population Health Sciences, Institute for Global Health, University College London, London, UK

## Abstract

Rates of sexually transmitted infections (STIs) continue to rise across the world and interventions are essential to reduce their incidence. Past and recent studies have indicated this may be achieved using doxycycline post-exposure prophylaxis (PEP) and this has sparked considerable interest in its use. However, many unanswered questions remain as to its long-term effects and particularly potentially negative impact on human microbiomes and antimicrobial resistance among STIs, other pathogens, and commensals. In this review, we discuss seven areas of concern pertaining to the widespread use of doxycycline PEP.

## Introduction

As rates of sexually transmitted infections (STIs) increase around the world, interventions are urgently needed to reduce their incidence and associated morbidity.^1^ Successful implementation of preventative interventions such as HIV pre-exposure prophylaxis (PrEP) has resulted in dramatic reductions in the incidence of HIV globally.^2^ This has led to studies that have assessed if doxycycline, taken prophylactically, pre- and/or post-sexual exposure, can reduce the incidence of bacterial STIs such as syphilis (aetiological agent: *Treponema pallidum* subspecies *pallidum*), chlamydia (*Chlamydia trachomatis*) and gonorrhoea (*Neisseria gonorrhoeae*).^3,4^

In 2015, the French IPERGAY trial by Molina *et al*.^3^ randomly assigned 232 men or transgender women who have sex with men (MSM or TGW) at high risk of acquiring HIV (condomless anal sex with >2 different partners during past 6 months) using HIV PrEP to take 200 mg of doxycycline (a broad-spectrum tetracycline antibiotic) within 24 h, but no later than 72 h after having sex (doxycycline post-exposure prophylaxis; PEP). This resulted in a 70%–73% lower incidence of chlamydia and syphilis infections but had no effect on *N. gonorrhoeae*. A recent DoxyPEP trial in the USA by Luetkemeyer *et al*.,^4^ reported similar reductions in STI incidence (66%) including a 55% lower incidence of gonorrhoea among 501 MSM or TGW who were counselled to take doxycycline after condomless sex with at least one male in the past 2 months and taking HIV PrEP. The difference in effect on gonorrhoea incidence between these two studies may be partly explained by the lower prevalence of gonococcal resistance to doxycycline in the USA (20%–30% resistance; https://www.cdc.gov/std/statistics/gisp-profiles/default.htm)^5^ compared with France (56% resistance)^6^ at the time of the trial, and that in DoxyPEP,^4^ participants were permitted to have more doses than in IPERGAY^3^ (seven versus three doses a week). Similar reductions in incident STIs (overall 65% and 55% for *N. gonorrhoeae*) were seen in the recent French DOXYVAC trial comparing 200 mg of doxycycline with Meningococcal B vaccine (4CMenB, Bexsero^®^) in 332 MSM on HIV PrEP^7^ and among MSM taking doxycycline daily (doxycycline PrEP) from a small pilot trial in 2011 from the USA (OR 0.3; 95% CI 0.08–1.09).^8^ However, due to the low sample size (*n* = 30) in the latter, doxycycline PrEP has not yet been recommended by public health organizations and, accordingly, this discussion will focus on doxycycline PEP.

The findings of these PEP studies and similar studies have led the San Francisco Public Health Unit to recommend doxycycline PEP to cis men and trans women who: (1) have had a bacterial STI in the past year; (2) report condomless anal or oral sexual contact with ≥1 cis male or trans female partner in the past year; and (3) those with a history of syphilis.^9^

Although the interim CDC statement in 2023 after the CROI conference does not advocate for or against doxycycline PEP, it has previously included a box in its HIV PrEP guidelines describing how this doxycycline PEP could be given if required.^10^ The USA International Antiviral Society in 2022,^11^ in contrast, states that pending further data on doxycycline’s effect on antimicrobial resistance (AMR) and the microbiome, the use of DoxyPEP should be considered on a case-by-case basis. In 2022, the British Association for Sexual Health and HIV and the UK Health Security Agency updated their guidance that they do not endorse the use of doxycycline taken as PEP or PrEP to prevent STIs.^12^ The WHO has not yet provided any strong statement advocating for or against its use.

In this targeted review, we reviewed the published literature regarding the effects of using doxycycline on AMR, specifically in the four most common bacterial STIs, other bacterial pathogens, and on the human microbiome as a proxy for what could occur in practice during widespread doxycycline PEP use. Where no direct evidence was available for doxycycline, we provide evidence from other antimicrobials or current AMR concerns. Using this evidence, we generated seven hypotheses pertaining to important concerns about the widespread use of doxycycline PEP.

## AMR

### AMR in STIs

#### AMR in N. gonorrhoeae

The doxycycline PEP randomized controlled clinical trials (RCTs)^3,4^ have only published results on the effects of doxycycline PEP on tetracycline resistance in *N. gonorrhoeae* and *C. trachomatis*. No statistically significant increase in doxycycline resistance was seen in either of the two RCTs that reported this outcome.^3,4^ The important caveats to this finding include the short time of follow-up and the very small number of *C. trachomatis* [*n* = 5 (IPERGAY)^3^] and *N. gonorrhoeae* isolates assessed [*n* = 9 (IPERGAY);^3^  *n* = 47 (DoxyPEP)].^4^ Notably, a substantial proportion of the *N. gonorrhoeae* isolates detected in the doxycycline arm of the DoxyPEP study were cultured from the oropharynx—a site where infection is mostly asymptomatic and where the risk is high for the acquisition of AMR through horizontal gene transfer of resistance determinants from commensal *Neisseria* species.^13^ Interim results presented at CROI 2023^14^ found that in each arm of the trial, two high-level tetracycline-resistant *N. gonorrhoeae* isolates were found, but in the DoxyPEP arm, there were an additional four low-level-resistant *N. gonorrhoeae* isolates (none in the control arm). The anatomical site from which these isolates were cultured from was not specified. Additional data from CROI 2023^14^ reported that the *N. gonorrhoeae* in the DoxyPEP arm had acquired resistance to a greater number of other antibiotics compared with those in the control arm. Among 20 *N. gonorrhoeae* isolates from DoxyPEP users, three were resistant to azithromycin, two to ciprofloxacin, and one to benzylpenicillin. In 19 non-PEP users, the only additional resistance found was to benzylpenicillin in two isolates. No isolate was resistant to ceftriaxone or cefixime, the drugs currently used to treat gonorrhoea. However low sample size limits inference of statistical significance.

While there was no significant increase in doxycycline-resistant commensal *Neisseria* in the DoxyPEP arm between baseline and Month 12 (63%–70%), at Month 12, however, there was significantly higher doxycycline resistance compared with control (70% versus 45%, *P* = 0.0017).^13^

Co-selection of resistance to other classes of antimicrobials is another possible concern for *N. gonorrhoeae* and other bacterial species.^15^ Many concerns are hypothetical, i.e. because we lack evidence that we hope will be gathered in many ongoing and future studies. Both the *tet*(M) gene and the *rpsJ* V57M mutation are strongly associated with gonococcal resistance and decreased susceptibility to doxycycline and selection by doxycycline use. However, also increased MtrCDE efflux is associated with doxycycline decreased susceptibility to doxycycline as well as to many other antimicrobials.^16^ Furthermore, both the *tet*(M) gene and the *rpsJ* V57M mutation may decrease susceptibility to other current but also future tetracycline derivates. Recent genomic studies of *N. gonorrhoeae* isolates have highlighted the potential for selecting cross-resistance to other antimicrobials with doxycycline use, especially when all tetracycline lineages are selected.^17,18^ Among MSM populations, the majority of *N. gonorrhoeae* isolates were found to have intermediate tetracycline MICs, which the authors warned could represent a reservoir for rapid evolution of resistance.^17^

#### AMR in C. trachomatis

No evidence of elevated doxycycline MICs was found in the five *C. trachomatis* isolates assessed in the IPERGAY study.^3^ The successful use of doxycycline for STIs for over five decades with little evidence of the emergence of resistance^19^ suggests it is a limited risk that doxycycline PEP selects *C. trachomatis* resistance to doxycycline. Nonetheless, the transfer of tetracycline resistance genes between *Chlamydia* species has been reported *in vitro* in pigs, and likely resulted from pigs being fed fish meal treated with tetracyclines.^20^ Similarly treatment failures with reduced susceptibility to tetracycline has been reported.^21–24^ In the DoxyPEP study,^4^  *C. trachomatis* incidence was reduced by 74%–88%. The emergence of doxycycline resistance in *C. trachomatis* would be serious as it is the most efficacious treatment for urogenital^25^ and rectal chlamydial infections,^26^ especially for lymphogranuloma venereum (LGV) infections.

#### AMR in Mycoplasma genitalium

Doxycycline is very important for empirical treatment of non-gonococcal urethritis and cervicitis and has emerged as an important component of sequential therapy for *M. genitalium*. In the sequential therapy, doxycycline is given for 7 days to cure 30%–40% of *M. genitalium* infections^27^ and reduce the *M. genitalium* bacterial load in many of the uncured *M. genitalium* infections and thereby facilitate the clearance by subsequent macrolide or fluoroquinolone therapy. Resistance to doxycycline in *M. genitalium* would reduce the effectiveness of doxycycline in this successful empiric as well as sequential treatment. In an IPERGAY sub-study, the authors reported mutations that have been previously associated with tetracycline resistance in other bacterial species in-vivo in participants who had taken doxycycline for treatment or prophylaxis, suggesting that the acquisition of these mutations may have been enhanced by the use of doxycycline.^28^ Another recent study has found that tetracycline resistance in *M. genitalium* can be caused by alterations in intrinsic efflux pumps resulting in increased MICs of doxycycline, tetracycline and minocycline.^29^ Intensive intermittent use of doxycycline, particularly in a high-prevalent MSM population on HIV PrEP, may thus reduce the efficacy of not only doxycycline but also one of the last resort *M. genitalium* treatments—minocycline.^30,31^

#### AMR in T. pallidum

Difficulties with culturing *T. pallidum* have made it difficult to evaluate tetracycline resistance in *T. pallidum*.^32^ There are, however, rare published reports of treatment failure following doxycycline therapy (*n* = 1),^33^ and resistance-conferring *tet*(B) genes were detected in a small proportion of *T. pallidum*-positive samples in a single study.^34^ However, contamination of these samples with *tet*(B) from another source cannot be excluded.^32^ Resistance to doxycycline could hypothetically emerge via acquisition of *tet*(B), but also mutations in ribosomal proteins or point mutations in the 16S rRNA gene. Whilst these 16S rRNA mutations have not been detected thus far, they are rarely screened for and they might emerge explosively, as was the case for the analogous 23S rRNA macrolide-resistance mutations whose prevalence increased from 0% to 90% in *T. pallidum* in some settings during only 10 years following intensive use of macrolides.^35,36^

In summary, for the treatment of STIs, while doxycycline is not recommended for treatment of *N. gonorrhoeae* due to the high rates of resistance, the potential risks for *C. trachomatis* where doxycycline remains the optimal treatment, *M. genitalium* where doxycycline is important for empirical treatment as well as in sequential treatment, and effects in regard to selection of cross-resistance to other tetracyclines and other classes of antimicrobials, enhanced phenotypic and molecular doxycycline resistance surveillance are urgently warranted, including tests of cure and genomic sequencing of STI pathogens from doxycycline treatment failures and individuals taking doxycycline PEP.

### AMR in other pathogenic bacterial species

The WHO has declared AMR as one of the greatest threats to global health that will cost an estimated US$3.4 trillion a year by 2030.^37^ It has been estimated that AMR infections resulted in 1.3–5.0 million deaths in 2019.^38^ The majority were caused by six pathogens, most of which can also be human commensals in different anatomical sites—namely *Escherichia coli*, *Staphylococcus aureus*, *Klebsiella pneumoniae*, *Streptococcus pneumoniae*, *Acinetobacter baumannii* and *Pseudomonas aeruginosa*. A number of studies have found that much of the AMR in these pathogens is caused by bystander selection, i.e. due to antimicrobials used for other indications.^39^ An example of this effect is the strong positive association between country-level penicillin consumption and reduced susceptibility to penicillin in *S. pneumoniae.*^40^ Further studies have suggested the need to keep population-level antimicrobial consumption below certain thresholds to prevent the emergence of AMR.^31^ To the best of our knowledge, no such threshold has been established for tetracyclines. Tetracyclines such as doxycycline do, however, also exert an evident bystander effect.^15^ A recent systematic review of the AMR selection by the use of oral tetracyclines on human flora^41^ reported increases in tetracycline-resistant *Streptococcus* strains in subgingival flora, *E. coli* in the gastrointestinal tract and a 5.8-fold increase in tetracycline-resistant respiratory tract pathogens. An RCT also found that the use of tetracycline for acne was associated with increased prevalence of tetracycline resistance in *E. coli* in the guts of patients and their relatives.^42^ Another RCT found that the receipt of 14 days of oral tetracycline resulted in an elevated prevalence of tetracycline resistance in subgingival bacteria and that this effect persisted for over 12 months.^43^ Interim data from CROI 2023^14^ reported an 8% absolute increase in doxycycline-resistant *S. aureus* in the DoxyPEP study^4^ arm (from 4% to 12%, *P* = 0.0008) (but not in the control arm) between baseline and Month 12. No change was seen for MRSA. Larger sample sizes will be required to evaluate if these increases are significant.

### AMR in commensal bacterial species

By virtue of their frequent ubiquitous presence and high bacterial loads, commensal bacterial are at the highest risk for bystander selection of AMR.^39^ This is of significant concern as selected chromosomal and plasmid-harboured AMR determinants can be subsequently transferred to pathogens via horizontal gene transfer mechanisms such as conjugation and transformation.^44^ Plasmids frequently contain genes that confer resistance to tetracyclines as well as other classes of antimicrobials. Intense doxycycline selection pressure could thus also increase the prevalence of resistance to other classes of antimicrobials.^15,45,46^ While tetracyclines are not recommended for the primary treatment of anaerobic infections, previous reports have shown that increased use of tetracyclines results in commensal anaerobic bacteria no longer responding to first- and second-generation tetracyclines.^47^

### Persistence, tolerance and AMR

AMR involves the inherited ability of a bacterial species to grow at high concentrations of an antimicrobial. As such it involves an increase in the MIC of the antimicrobial for the microbe.^48^ Tolerance, in contrast, refers to the ability to survive transient exposure to high concentrations of antimicrobials without an increase in antimicrobial MIC. Tolerance is typically acquired by slowing down several bacterial metabolic processes.^48^ Transient exposures to high *in vitro* concentrations of antimicrobials, including tetracyclines, has been shown to induce tolerance in all bacterial species studied thus far, including *N. gonorrhoeae*.^49^ Bacterial tolerance has also been linked to persistent and recurrent infections with *E. coli* and *P. aeruginosa.*^50^ Tolerance has also been shown to play a key role in the pathway toward the genesis of AMR in a number of bacteria.^48,50^

### Off-target effects of tetracyclines

In many bacterial species, tetracyclines at therapeutic and subtherapeutic doses can induce or select resistance to other classes of antimicrobials through co-localization of AMR genes on chromosomes or plasmids, enhanced activity of efflux pumps^51^ and other mechanisms.^52^ In addition, tetracyclines can have antimetalloprotease and possibly anti-inflammatory activity in humans.^52^ Several studies have found an increased risk of various infections following long-term antibiotic (mainly tetracycline) use for acne [upper respiratory tract infection (OR 2.8, 95% CI 2.4–3.2), urinary tract infection in women (OR 1.9, 95% CI 1.4–2.5) and pharyngitis (OR 4.3, 95% CI 1.5–12.5)].^53^ This effect may be mediated by reduced colonization resistance for the pathogens. Accordingly, tetracyclines may reduce the abundance of commensal bacteria, which normally protect their host from initial colonization and subsequent infection by the pathogen.^54^ Doxycycline can cause photosensitivity in response to UV radiation, an effect that is mediated by doxycycline-induced oxidative stress and mitochondrial toxicity.^55^ Finally, a range of rare adverse events experienced by patients taking doxycycline have been noted to improve or resolve following doxycycline discontinuation, such as benign intracranial hypertension and oesophagitis.^55^ These uncommon adverse events were reflected in the low discontinuation rates in the recent doxycycline trials—0.9% and 2.0% in the DOXYVAC^7^ and DoxyPEP^4^ trials, respectively. In the DoxyPEP trial, five participants had an adverse event possibly related to doxycycline, while in the DOXYVAC trial, drug-related adverse events occurred in 5.7% taking doxycycline and 34.6% with 4CMenB.

## Altered microbiome

The effects of antimicrobials on the microbiome, which frequently protects us from infections and diseases, are extremely complex. In particular, much remains to be learnt about the clinical significance of antimicrobial-induced changes in the microbiome. Recent studies have, for example, shown that a number of classes of antimicrobials have a dose-dependent effect on increasing the risk for a range of conditions such as obesity, allergy, asthma, inflammatory bowel disease, coeliac disease and attention-deficit hyperactivity disorder (ADHD).^56^ This effect may persist for decades after the cessation of the antimicrobial and may even be transferred transgenerationally—presumably both the effect and transfer are associated with the microbiome.^56^ Whilst tetracyclines appear to be one of the safer classes of antimicrobials for the gut microbiome,^57^ a number of studies have found that they can induce large perturbations in the microbiome. For example, one RCT found that doxycycline taken for 10 days resulted in reduced gut bacterial diversity including a 100-fold reduction in bifidobacteria, as well as increased prevalence of tetracycline resistance in gut commensals.^58^

The effects on the microbiome may be even more complex in those living with HIV, who show an altered microbiome compared with those who are HIV negative.^59^ Lastly, tetracyclines have been reported to have an effect on the gut microbiomes of both humans and food animals such as pigs, chickens and cows.^60–62^

Further evidence of clinically relevant effects on the microbiome come from studies that reveal that tetracycline intensifies the blood-thinning effect of anticoagulant medication (via reducing the abundance/activity of bacteria that produce vitamin K).^55^ Studies have found that the concomitant consumption of doxycycline with acenocoumarol or phenprocoumon increases the risk of bleeding by approximately 2.5-fold.^63^

## Arrested immunity and loss of colonization resistance

Human studies have theorized that if STIs are treated before immunity is developed to the infection, then the risk of reinfection can be higher due to the loss of any temporary and/or partial immunity. A study following up women with genital *C. trachomatis* infection found that those treated with antimicrobials had a 4-fold higher probability of reinfection than women in whom the infection was cleared by their immune system.^64^ The authors concluded that treatment for chlamydia may attenuate protective immunity in some patients. Similar findings have been found for syphilis.^65^ In the case of chlamydia, this arrested immunity is a plausible explanation for the paradoxical increase in chlamydia incidence in populations that have experienced intensive screening and treatment.^66,67^ It is possible that intermittent doxycycline use will reduce individual and population partial immunity to *C. trachomatis* as well several other STI agents and pathogens and thus paradoxically increase the risk of reinfection. It is, however, important to note that the major doxycycline PEP studies have not found any evidence of arrested immunity but only reduced incidence (including reinfections) of various STIs.

## Difficulties in syphilis diagnosis and treatment

Doxycycline is active against *T. pallidum*; however, because 14–28 day treatments are needed to effectively cure many syphilis cases, doxycycline PEP may fail to cure a proportion of incident syphilis cases. This problem will be aggravated by the fact that in the contemporary syphilis epidemics, a substantial proportion of all the cases of syphilis can be repeat episodes.^68^ Syphilis reinfections are typically asymptomatic and detected only by detecting a two-titre increase in non-treponemal tests.^69,70^ Hypothetically, this detection could prove difficult for healthcare practitioners in a number of settings^68^ such as where only treponemal tests (rapid point-of-care tests) are used. It remains unknown how the intermittent use of doxycycline will affect the clinical and serological progression of syphilis. We theorize that if someone uses doxycycline PEP a week after reinfection with *T. pallidum*, this may limit the increase in non-treponemal titre to a maximum of one titre instead of two titres if no doxycycline has been taken. This would mean that this repeat episode of syphilis would not be diagnosed at this visit. In this circumstance the growth and dissemination of *T. pallidum* could be delayed and missed. Because *T. pallidum* is known to disseminate and invade the CNS early after infection, it is also possible that neurosyphilis infections will be masked and their detection delayed.

## Historical examples of chemoprophylaxis were halted due to AMR

Chemoprophylaxis pre- and post-sex have been used since World War I in attempts to reduce the incidence of STIs.^71–76^ Although many of these studies used historical control groups, the initial studies reported impressive reductions in the incidence of STIs such as gonorrhoea, syphilis and chancroid.^72^ As with the recent results of the doxycycline PEP trials, these positive findings generated considerable enthusiasm for chemoprophylaxis.^73^ However, the widespread use of antimicrobials was followed by the emergence of AMR. In one such study, the use of minocycline PEP to prevent *N. gonorrhoeae* infection was noted to be efficacious against susceptible *N. gonorrhoeae* isolates but had no effect against resistant isolates.^73^ It was concluded that extensive use of minocycline would ‘have limited effectiveness as a public-health measure because of the tendency to select resistant gonococci.’ These insights curbed the enthusiasm for these interventions.^73^

## Doxycycline PEP results in high doxycycline exposure in a subset of users

In the DoxyPEP study,^4^ while participants were permitted to take one dose per day, the median number of doses taken per month was 4 based on self-reporting, which is similar to that used in the IPERGAY trial where participants took a median of 6.8 pills (680 mg) per month (about three 200 mg doses).^3^ However, in the DoxyPEP trial, 25% took 10 or more doses (≥2 g per month).^4^ These doses are higher than the 1.4 g (100 mg twice a day for 7 days) used to treat chlamydia. Therefore for those where chlamydia is the sole infection, the preventative doxycycline PEP consumption was higher than if these participants were treated monthly for chlamydia.

The only organization that currently recommends the use of PEP (the San Francisco Public Health Unit) follows the DoxyPEP study^4^ protocol that allows the very high level consumption of 200 mg of doxycycline per day.^9^ Of note, the San Francisco guidelines continuing recommending 3 monthly STI screening in those using doxycycline, which may question the efficacious utility of doxycycline use between screens.

## Rollout of doxycycline PEP and the precautionary principle

If public health agencies advocate for the use of doxycycline PEP beyond controlled research settings this implies that they have concluded that sufficient evidence is available and the benefits outweigh the risks and potential future harms. Understandably, the lay public will consider this means that doxycycline PEP is evidently safe and its use will likely increase dramatically among MSM but also additional populations. It will be very hard to reverse this trend and we cannot exclude that additional therapeutic antimicrobials will also be in the pipeline for PEP (or PrEP), i.e. prescribed by clinicians or purchased online without a prescription (see below). This would significantly challenge a lot of the work performed to improve antimicrobial stewardship globally.

## Limitations

We did not use a systematic review methodology. In addition, interpretation of published evidence is often limited by small sample sizes and few isolates tested for AMR, both in the doxycycline PEP studies^3,4^ and in the published literature. This makes drawing robust conclusions difficult. Ongoing surveillance of doxycycline PEP studies will hopefully provide more definitive evidence. Given these issues, we cannot exclude any biases arising from the limited evidence currently available and cognitive biases in our interpretation of this evidence.

## Important counter arguments

In addition to the proven efficacy of doxycycline PEP, we acknowledge a number of other counter arguments and where doxycycline PEP use could provide benefits.

Firstly, it has been estimated that approximately 8%–10% of people using HIV PrEP have used doxycycline prophylaxis from surveys of clients at London, Melbourne and Amsterdam sexual health clinics.^74–76^ A reasonable argument could be made that it would be preferable for these individuals to take their doxycycline under supervision. This is of particular concern given the small number of individuals who reported using antibiotics other than doxycycline such as amoxicillin,^75^ azithromycin^77^ or ciprofloxacin^76^ as PEP in studies undertaken prior to the recent doxycycline PEP trials,^3,4^ i.e. previous studies in the UK (*n* = 107 from 2018),^73^ Belgium (*n* = 187 from 2022)^75^ and the Netherlands (*n* = 321 from 2018),^74^ respectively. Many of these antibiotics were purchased online or were leftovers.^77^ Online purchase of doxycycline without a prescription is also possible. A popular international website, for example, has since 2018 offered online links to pharmacies that provide access to HIV PreP and doxycycline with or without a prescription (https://www.purchase-prep.com/). Secondly, if doxycycline could be targeted to the individuals most central in the sexual network, this could have a dramatic effect on the incidence of *C. trachomatis*, *T. pallidum* and possibly *N. gonorrhoeae* infections, at least in some countries. In Australia, for example, it has been estimated that 22 730 individuals have been prescribed HIV PrEP, with a small core group of 6% of these individuals accounting for 36% of all STIs.^78^ Targeting this group of 6% may have a large effect on STI incidence. Similarly, in the IPERGAY study, analysis among 429 MSM reported 39% of participants accounted for 86% of STIs.^79^ Analysis of 10 500 electronic health records by Traeger *et al*.^80^ explored 10 hypothetical doxycycline PEP-prescribing strategies and suggested that prescribing doxycycline PEP for a period of 12 months to people with an STI diagnosis, regardless of HIV PrEP status, could avert 42% of all subsequent STIs (while reducing the number of prescriptions by 59%). Similarly, targeting those with a syphilis diagnosis would avert 14% of all STIs and reduce prescriptions by 91%. Thirdly, by reducing the incidence of these STIs, targeted doxycycline PEP could also reduce the consumption of ceftriaxone and possibly macrolides in this population.^14^ Fourthly, proponents of doxycycline chemoprophylaxis argue that doxycycline is already used widely for the management of acne, malaria prophylaxis, skin and soft tissue and other infections. Whilst this is true, as reviewed above, this use has resulted in the emergence of AMR in a range of bacterial species as well as other problems. Lastly, some users and prescribers have reported that doxycycline PEP can empower users to take control of their sexual health. They report that a lower STI incidence may reduce their distress, thereby making sex more enjoyable for them.

In summary, doxycycline PEP is a novel intervention that may reduce the incidence of STIs, particularly of some STIs among high-risk MSM or TGW on HIV PrEP and MSM/TGW who are HIV positive. These findings are exceedingly exciting. However, as detailed above, many unknowns remain regarding: the long-term effects of intermittent, frequently high-consumption, doxycycline use (in PEP and PrEP), and likely future use of additional antimicrobials, and especially potentially negative impact on human microbiomes; selection of AMR and/or antimicrobial tolerance in STI agents, other pathogens and commensals; doxycycline off-target effects; effects on arrested immunity and colonization resistance; and effective messages concerning STI prevention and antimicrobial stewardship. Based on the need for continued collection of evidence regarding broader and long-term consequences, we consider it would be more prudent to follow the precautionary principle and provide doxycycline PEP in controlled settings, such as prioritizing prescriptions targeted at those with a recent STI combined with ongoing AMR surveillance for STIs, other pathogens and commensals. More attention should be placed also on the development of STI vaccines,^81,82^ and increased access to testing of symptomatic STI cases, including increased implementation of websites providing access to self-sampling testing kits^83^ or to pathology centres^84^ and the development of rapid, accurate point-of-care tests (detecting STIs plus AMR determinants).^85^ Finally, it is clear that people are already taking doxycycline for STI prophylaxis, some purchasing this online, and it is important that clinicians are fully informed of the potential risks to users and the possible population effects from AMR.
